# Reply to Harsanyi et al. Comment on “Hasan et al. Clinico-Pathological Features and Immunohistochemical Comparison of p16, p53, and Ki-67 Expression in Muscle-Invasive and Non-Muscle-Invasive Conventional Urothelial Bladder Carcinoma. *Clin. Pract.* 2023, *13*, 806–819”

**DOI:** 10.3390/clinpract16010010

**Published:** 2025-12-31

**Authors:** Abdulkarim Hasan, Yasien Mohammed, Mostafa Basiony, Mehenaz Hanbazazh, Abdulhadi Samman, Mohamed Fayek Abdelaleem, Mohamed Nasr, Hesham Abozeid, Hassan Ismail Mohamed, Mahmoud Faisal, Eslam Mohamed, Diaa Ashmawy, Mohamed Tharwat, Deaa Fekri Morsi, Abeer Said Farag, Eman Mohamed Ahmed, Noha M. Aly, Hala E. Abdel-Hamied, Doaa E. A. Salama, Essam Mandour

**Affiliations:** 1 Pathology Department, Faculty of Medicine, Al-Azhar University, Cairo 11884, Egypt; 2 Basic Medical Sciences Department, College of Medicine, University of Jeddah, Jeddah 23218, Saudi Arabia; 3 Preventive Medicine, Ministry of Health, Cairo 11516, Egypt; 4 Histology Department, Faculty of Medicine, Al-Azhar University, Cairo 11884, Egypt; 5 Urology Department, Faculty of Medicine, Al-Azhar University, Cairo 11884, Egypt; 6 Urology Department, King Abdullah Medical City, Makkah 24246, Saudi Arabia; 7 Clinical Oncology Department, Faculty of Medicine, Al-Azhar University, Cairo 11884, Egypt; 8 Pathology Department, Faculty of Medicine, Al-Azhar University, Damietta 34517, Egypt; 9 Pathology Department, Faculty of Medicine, Al-Azhar University, Assiut 71524, Egypt; 10 Pathology Department, Faculty of Medicine, Helwan University, Helwan 11795, Egypt; 11 Pathology Department, Faculty of Medicine for Girls, Al-Azhar University, Cairo 11884, Egypt; 12 Pathology Department, School of Medicine, Badr University in Cairo (BUC), Cairo 11829, Egypt

We would like to thank Harsanyi et al. for their thoughtful and constructive commentary on our recent article [1,2]. Their study of a large patient cohort provides valuable evidence regarding the prognostic roles of p53 and Ki-67, and we appreciate their contribution to this important discussion.

 **1.** **Cut-off Thresholds for p53 and Ki-67**

We predefined a p53 cut-off of 20% and a Ki-67 cut-off of 13%. Using these thresholds, and within the constraints of our sample size, p53 did not show a significant association with grade or stage, whereas Ki-67 was higher in muscle-invasive than non-muscle-invasive tumors and aligned with histologic grade. Accordingly, our inferences are limited to the a priori thresholds and study design. We also commend the commenters for the scope and rigor of their large cohort and for the valuable findings they report, which meaningfully advance discussion on threshold selection.

The commenters propose higher thresholds (e.g., p53 ≥ 40%, Ki-67 ≥ 30%) to increase specificity for stage and grade stratification (e.g., NMIBC vs. MIBC; low vs. high grade). We agree that altering the cut-off inevitably shifts the specificity–sensitivity balance and that between-study differences in preanalytical handling, analytical methodology (antibody clone/platform, detection system), interpretation criteria (scoring schema and thresholds), and endpoint definitions can yield divergent results even when the same biomarkers are assessed. Because our analysis plan centered on predefined thresholds, any re-analysis at alternative values would be exploratory and should be interpreted as such.

 **2.** **Positioning our thresholds within the literature**

Cut-offs for IHC markers are not universal; they vary with tumor type, cohort characteristics, assay conditions, and the clinical question being addressed. In urothelial carcinoma, Şentürk et al. [3] applied Ki-67 ≥ 13% and p53 ≥ 20% and demonstrated associations with stage and grade in bladder tumors, providing a precedent for thresholds in this range within UC cohorts. In other disease sites, for example, breast cancer, Soliman and Yussif [4] reported using a Ki-67 index of ~15% for risk stratification in defined contexts, underscoring that “optimal” values are context-dependent rather than fixed. Taken together, these examples support our decision to interpret our results strictly within our predefined thresholds while acknowledging that alternative cut-offs, particularly in larger cohorts and under different preanalytical/analytical conditions, may yield different operating characteristics.

 **3.** **Conclusions**

We are grateful for Harsanyi et al.’s engagement with our work. We believe that such scientific dialog advances the field, and we fully support future collaborative efforts aimed at standardizing immunohistochemical evaluation in urothelial carcinoma.

## References

[1] Harsanyi S., Novakova Z.V., Ziaran S., Danisovic L., Bevizova K. (2026). Comment on Hasan et al. Clinico-Pathological Features and Immunohistochemical Comparison of p16, p53, and Ki-67 Expression in Muscle-Invasive and Non-Muscle-Invasive Conventional Urothelial Bladder Carcinoma. *Clin. Pract.* 2023, *13*, 806–819. Clin. Pract..

[2] Hasan A., Mohammed Y., Basiony M., Hanbazazh M., Samman A., Abdelaleem M.F., Nasr M., Abozeid H., Mohamed H.I., Faisal M. (2023). Clinico-Pathological Features and Immunohistochemical Comparison of p16, p53, and Ki-67 Expression in Muscle-Invasive and Non-Muscle-Invasive Conventional Urothelial Bladder Carcinoma. Clin. Pract..

[3] Senturk N., Aybek Z., Duzcan E. (2010). Ki-67, p53, bcl-2 and bax expression in urothelial carcinomas of urinary bladder. Turk. J. Pathol..

[4] Soliman N.A., Yussif S.M. (2016). Ki-67 as a prognostic marker according to breast cancer molecular subtype. Cancer Biol. Med..

